# Theranostic vNAR-Based Immunoconjugates Achieve Selective Intracellular Cisplatin Delivery in Embedded 3D HER2-Positive Breast Cancer In Vitro Model

**DOI:** 10.3390/ph19040633

**Published:** 2026-04-17

**Authors:** Andrea C. Alfonseca-Ladrón de Guevara, Alejandro Manzanares-Guzmán, Jessica A. Badillo-Mata, Mirna Burciaga-Flores, Pavel H. Lugo-Fabres, Tanya A. Camacho-Villegas

**Affiliations:** 1 Unidad de Biotecnología Médica y Farmacéutica, Centro de Investigación y Asistencia en Tecnología y Diseño del Estado de Jalisco (CIATEJ), Guadalajara 44270, Jalisco, Mexico; andrea.alfoseca@gmail.com (A.C.A.-L.d.G.); almanzanares90@gmail.com (A.M.-G.); jebadillo_al@ciatej.edu.mx (J.A.B.-M.); 2 SECIHTI—Departamento de Modelación de Nanomateriales, Centro de Nanociencias y Nanotecnología, Universidad Nacional Autónoma de México (CNYN-UNAM), Carretera Tijuana—Ensenada km 107, Ensenada 22860, Baja California, Mexico; mirna.b.flores@ens.cnyn.unam.mx; 3 SECIHTI—Unidad de Biotecnología Médica y Farmacéutica, Centro de Investigación y Asistencia en Tecnología y Diseño del Estado de Jalisco (CIATEJ), Guadalajara 44270, Jalisco, Mexico; plugo@ciatej.mx

**Keywords:** shark vNAR, EGFRvIII-targeted therapeutics, HER2-positive breast cancer, drug delivery, 3D spheroid models, next-generation immunoconjugates, theranostics

## Abstract

**Background/Objectives**: Precise intracellular delivery of chemotherapeutics remains a major challenge in HER2-positive breast cancer, where intratumoral heterogeneity and limited tissue penetration constrain efficacy. A key contributor is the tumor-restricted epidermal growth factor receptor variant III (EGFRvIII), a constitutively active, ligand-independent mutant generated by deletion of exons 2–7. Although classically associated with glioblastoma, lung (NSCLC), head/neck, and prostate cancers, EGFRvIII is also present in subsets of HER2-positive breast cancers, where low-abundance subclones drive aggressive phenotypes and attenuate therapeutic responses. HER2–EGFRvIII co-expression amplifies oncogenic signaling, supported by frequent co-expression in ErbB2-positive primary tumors and metastases, and by sustained receptor phosphorylation in the absence of EGFR gene amplification, depicting EGFRvIII as a compelling therapeutic target. **Methods**: We evaluated the shark-derived single-domain antibody vNAR R426 as a modular theranostic platform for receptor-mediated cisplatin delivery. Conjugation to cisplatin and fluorescein enabled simultaneous intracellular drug transport and immunofluorescence-based detection in EGFRvIII-positive SKBR3 cells and 3D spheroids. The compact vNAR-based immunoconjugates support efficient receptor recognition, internalization, and intracellular trafficking, features rarely achieved by conventional IgG antibodies. **Results**: vNAR_CDDP_ elicited robust, receptor-mediated cytotoxicity, achieving an IC_50_ of 2.68 µM—approximately 50-fold lower than that of free cisplatin—while unconjugated vNAR maintained scaffold biocompatibility. In three-dimensional spheroid models, the theranostic vNAR (vNAR_CDDP+FITC_) exhibited deep and uniform penetration throughout tumor-like architectures, with immunofluorescence intensity closely correlating with regions of intracellular drug delivery and the initiation of cytotoxic responses. Notably, cisplatin conjugation did not impair tissue diffusion or receptor engagement, facilitating effective payload delivery to both peripheral and central cell populations. **Conclusions**: By integrating tumor-restricted targeting and efficient intracellular drug delivery within a modular single-domain scaffold, vNAR R426 represents a next-generation theranostic platform capable of addressing intratumoral heterogeneity. This approach combines potent cytotoxic activity with immunofluorescence-based detection, thereby advancing the rational design of precision therapeutics for HER2-positive breast cancer.

## 1. Introduction

HER2-positive breast cancer, defined by amplification and overexpression of the HER2 oncogene, represents 15–20% of all breast malignancies and drives uncontrolled proliferation, survival, and metastasis [1]. Despite substantial clinical gains achieved with HER2-targeted therapies—including Trastuzumab, Pertuzumab, and antibody–drug conjugates (ADCs)—up to half of patients develop resistance or relapse, highlighting the imperative need for strategies that overcome intratumoral heterogeneity and HER2-independent survival pathways [2].

Resistance to HER2-targeted therapies arises through HER2 truncation, compensatory PI3K–AKT activation, and the upregulation of alternative ErbB receptors—most notably EGFR—, whose crosstalk with HER2 redirects oncogenic signaling under therapeutic pressure. In parallel, HER2 overexpression promotes robust homodimer and heterodimer formation across the EGFR family, diversifying downstream signaling programs that sustain tumorigenesis [2,3]. These observations underscore the necessity of novel approaches capable of targeting subclonal populations within heterogeneous tumors. A defining axis of this heterogeneity is the tumor-restricted EGFR variant III (EGFRvIII), a constitutively active, ligand-independent mutant receptor resulting from the deletion of exons 2–7 [4,5,6]. Although classically linked to glioblastoma, EGFRvIII has also been detected in subsets of HER2^+^ breast cancers, where low-abundance subclones drive aggressive behavior and attenuate therapeutic responses [2,7,8,9].

The HER2 receptor can directly interact with EGFRvIII, amplifying downstream signaling and enhancing tumorigenesis. This is supported by the high frequency of EGFRvIII co-expression in ErbB2-positive primary tumors (~40%) and metastatic lymph nodes (~75%). Notably, EGFRvIII remains phosphorylated in breast carcinoma cells and sustains constitutive, prolonged signaling compared with EGFR–HER2 complexes, even in the absence of EGFR gene amplification [7,8]. These interactions collectively potentiate migration, invasion, and tumor progression. Further, EGFRvIII’s absence from normal tissue and its structural divergence from wild-type EGFR (wtEGFR) make it a compelling, tumor-restricted target. Selective elimination of EGFRvIII-expressing cells represents a rational strategy to prevent recurrence driven by resistant subpopulations. Conventional tyrosine kinase inhibitors or anti-EGFR monoclonal antibodies inadequately address this limitation. These properties make EGFRvIII a uniquely actionable target within HER2-driven tumors.

Single-domain antibodies, including shark-derived variable New Antigen Receptors (vNARs), offer unique advantages for targeted therapy [10,11,12,13]. With a molecular weight of 12–15 kDa, vNARs represent the smallest naturally occurring antigen-binding domains, combining high solubility and stability with the capacity to access cryptic or conformationally constrained epitopes [14,15,16]. The reduced molecular size of vNARs enhances tumor penetration and facilitates modular conjugation with therapeutic payloads, making them an optimal scaffold for the development of next-generation immunoconjugates. Notably, vNARs have been successfully employed to target tumor-specific variants such as EGFRvIII, in which precise recognition and efficient internalization are essential for therapeutic efficacy [17].

We previously reported the isolation of the first anti-EGFRvIII vNAR (vNAR R426) from a non-immune *Potamotrygon* spp. library and demonstrated that vNAR R426 mediates EGFRvIII-specific endocytosis, nuclear retrotransport via Coat Protein Complex I (COPI), and targeted intracellular delivery of cisplatin in EGFRvIII-positive glioblastoma U87-MG cells, resulting in a ten-fold increase in cytotoxic potency relative to free cisplatin [17]. vNAR R426 exhibited high selectivity for EGFRvIII over wild-type EGFR, thereby minimizing off-target toxicity and expanding the therapeutic window—attributes that are particularly advantageous in HER2-positive tumors, where EGFRvIII-positive resistant subclones may persist despite HER2-targeted therapies.

Three-dimensional (3D) culture systems provide a physiologically relevant framework for modeling tumor architecture, preserving cell–cell and cell–extracellular matrix interactions that are lost in conventional monolayer cultures [18,19,20]. Spheroid-based models reproduce oxygen and nutrient gradients, yielding proliferative outer layers and hypoxic, therapy-resistant cores that mirror intratumoral heterogeneity in vivo [21,22,23,24]. Such models enable rigorous assessment of penetration, uptake, and efficacy of targeted therapeutics in a spatially relevant context.

In this study, we further characterized the anti-EGFRvIII vNAR R426 in the human adenocarcinoma SKBR3 cell line, evaluating both cisplatin delivery (vNAR_CDDP_) and imaging using a fluorescein-conjugated vNAR (vNAR_FITC_). vNAR_CDDP_ exhibited high specificity for EGFRvIII and efficient receptor-mediated internalization, delivering cisplatin effectively at concentrations nearly 50-fold lower than free drug, and inducing robust cytotoxicity in both monolayer and 3D spheroid cultures. The unconjugated vNAR R426 displayed no intrinsic cytotoxicity, confirming scaffold biocompatibility.

Importantly, both vNAR_FITC_ and the theranostic vNAR immunoconjugate (vNAR_CDDP+FITC_) exhibited efficient penetration and robust fluorescent labeling throughout three-dimensional SKBR3 spheroids, with only modest attenuation observed toward central necrotic regions. These results indicate that cisplatin conjugation does not impair tissue diffusion or receptor engagement, thereby establishing a direct association between intratumoral distribution and therapeutic efficacy. The integration of spatial immunofluorescence analysis with functional viability assessments offers a mechanistic framework for evaluating drug delivery and cytotoxic potency in complex tumor models.

Collectively, these results highlight the promise of vNAR-based theranostic platforms for precision therapy in HER2-positive breast cancers harboring EGFRvIII-positive subclones, offering both deep tumor penetration and potent, targeted cytotoxicity in physiologically relevant 3D models.

## 2. Results

### 2.1. Recombinant vNAR R426 as a Modular Platform for EGFRvIII-Targeted Imaging and Cisplatin Delivery

**Figure 1:**
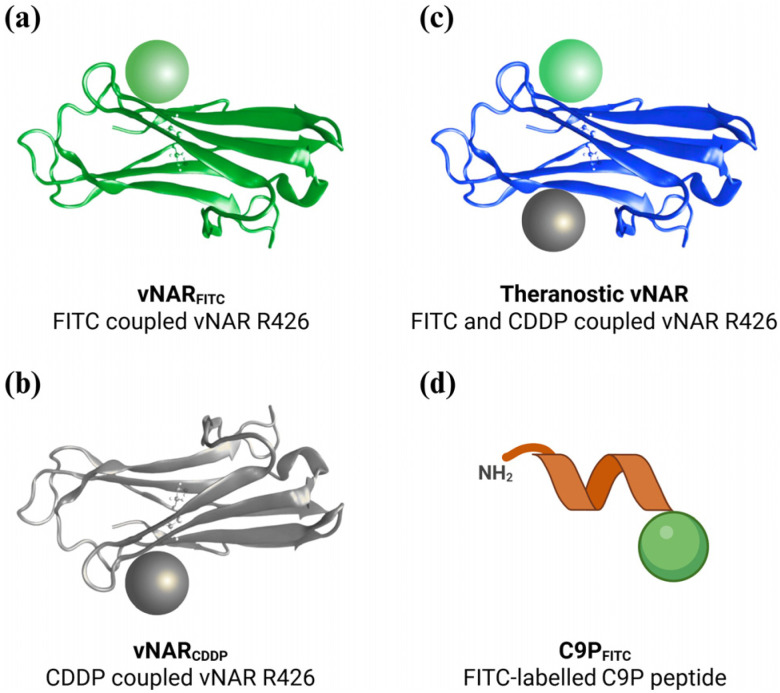
Design and generation of immunoconjugates and theranostic immunoconjugate based on variable new antigen receptors (vNAR), small variable single domains derived from sharks. (**a**,**b**) Schematic representation of the vNAR R426 immunoconjugates that are functionalized either with fluorescein (Immunofluorescent detection, called vNAR_FITC_) or with the chemotherapy drug cisplatin (Targeted drug delivery, called vNAR_CDDP_). (**c**) Design of a theranostic vNAR immunoconjugate that conjugates both cisplatin and fluorescein, combining therapy and imaging functions in a single molecule (vNAR_CDDP+FITC_). (**d**) Fluorescein-tagged C9P peptide, used as a comparison control targeting the HER2 protein (C9P_FITC_). Panels (**a**–**c**) outline the stepwise assembly process and multifunctional potential of the vNAR R426 scaffold for precision targeting of cell surface EGFRvIII receptors. Gray spheres represent cisplatin (CDDP), green spheres represent FITC. Created in BioRender. Lugo, P. (2026) https://BioRender.com/vfhwcti (accessed on 5 February 2026).

We previously isolated the first single-domain anti-EGFRvIII antibody from a non-immune freshwater stingray library (vNAR R426). This high-affinity, EGFRvIII-specific binder was validated both in vitro and in silico. The vNAR R426 enabled efficient receptor-mediated endocytosis and COPI-dependent nuclear trafficking, which may significantly enhance nuclear delivery of cisplatin upon conjugation and culminate in cytotoxicity that surpasses the free chemotherapy drug [17]. We expressed the recombinant vNAR R426, purified it via Ni-NTA chromatography, and confirmed its specific binding to EGFRvIII via recognition ELISA and identity by Western blot before conjugating it with fluorescein-5-isothiocyanate (FITC) for immunofluorescence detection, cisplatin (CDDP) for targeted therapy or both for assessment of theranostic vNAR R426 platform, following our previous methodology [17]. This workflow demonstrates that vNAR R426 is a versatile, modular platform for simultaneous imaging and cytotoxic delivery in EGFRvIII-expressing cells, highlighting its promise as a next-generation theranostic minimal scaffold (Figure 1a–c). Fluorescein-tagged C9P peptide was employed as a comparison control targeting the HER2 protein (C9P_FITC_) (Figure 1d).

The specificity and functional potential of the shark-derived vNAR R426 were evaluated through a series of complementary biochemical and biophysical assays, as detailed in the Supplementary Data. Statistical significance (** *p* < 0.0016) was determined between the recognition of pEGFRvIII compared to wtEGFR when the vNAR R426 was used. A similar result was observed with the commercial monoclonal antibody targeting the pEGFRvIII epitope (** *p* < 0.0026). Both the antibody and the single domain were selected using the LEEKKGNYVVTDH epitope. This epitope is unique to EGFRvIII (Figure S1). These findings confirm that vNAR R426 selectively engages the EGFRvIII mutant receptor, a critical feature for targeted cytotoxicity and theranostic applications.

Western blot analysis of heat-shock lysates from SKBR3 (EGFRvIII^+^/wtEGFR^+^/HER2^+^) breast cancer cells and U87-MG (EGFRvIII^+^/wtEGFR^+^/HER2^−^) glioblastoma cells confirmed the specificity of vNAR R426. vNAR R426 recognized both the monomeric form of EGFRvIII (145 kDa) and a lower-molecular-weight lysate-associated form (~45 kDa). In contrast, the commercial monoclonal antibody detected only bands at approximately 50 and 80 kDa, likely corresponding to EGFRvIII truncation variants (Figure S2). Collectively, these results validate vNAR R426 as a selective molecular scaffold that distinguishes EGFRvIII from wild-type receptors, thereby enabling precise targeting in heterogeneous HER2-positive tumor contexts while sparing wild-type EGFR.

To evaluate the potential of vNAR R426 as a cytotoxic delivery carrier, cisplatin was conjugated to the vNAR R426, forming vNAR_CDDP_. Ultraviolet–visible (UV–Vis) spectral analysis revealed spectral modifications indicative of the formation of a stable drug–protein complex (Table S1). Area under the curve (AUC) analysis in the 190–220 nm range yielded an AUC of 17.12 for vNAR_CDDP_ versus 23.04 for Free cisplatin, reflecting successful conjugation (Table S2). The drug-to-protein ratio (DAR) was calculated at ~1.34, which is favorable given the compact size of vNAR and potential steric constraints. Conjugation efficiency was estimated at 67.5%, based on the assumption of two hypothetical conjugation sites per vNAR molecule. This stoichiometry is anticipated to optimize cellular penetration and nuclear delivery.

Together, these findings demonstrate that vNAR R426 maintains target specificity following drug conjugation and facilitates efficient cytotoxic payload attachment. These results support the potential utility of vNAR R426 for therapeutic and theranostic applications in tumor contexts characterized by low EGFRvIII expression and HER2 overexpression.

### 2.2. vNAR R426 Internalizes via Receptor-Mediated Endocytosis in EGFRvIII-Positive Cells

**Figure 2:**
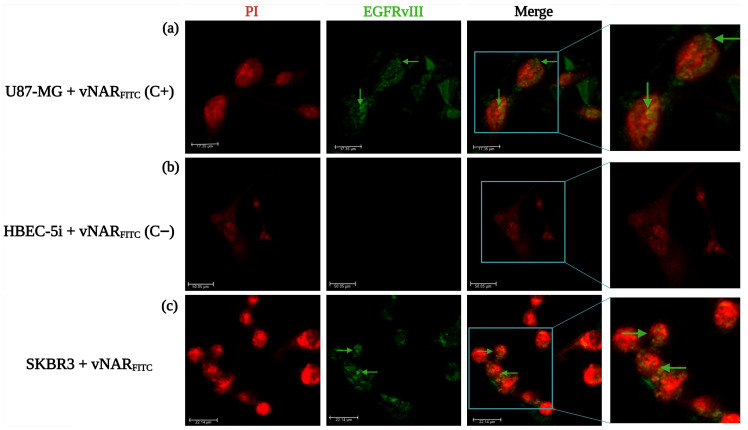
Confocal microscopy analysis of vNAR_FITC_ binding and intracellular uptake was conducted via immunofluorescence using three cell lines with defined molecular profiles: (**a**) U87-MG cells (EGFRvIII^+^/wtEGFR^+^/HER2^+^) served as the positive control for EGFRvIII recognition; (**b**) HBEC-5i cells (EGFRvIII^−^/wtEGFR^+^/HER2^−^) served as the negative control for EGFRvIII and HER2, and as the positive control for wtEGFR expression; and (**c**) SKBR3 cells (EGFRvIII^+^/wtEGFR^+^/HER2^+^) were used to assess vNAR_FITC_ specificity for EGFRvIII in the context of co-expression of all three markers. Cells were incubated with vNAR_FITC_ (0.081 µM) for 24 h. Immunofluorescence corresponding to vNAR_FITC_ is indicated by green arrows, and nuclei were counterstained with propidium iodide (red). Images were acquired at 40× magnification. Scale bars: 17.35 µm (**a**), 50.5 µm (**b**), 22.14 µm (**c**). All experiments were performed in triplicate.

To define its cellular binding profile, FITC-labeled vNAR R426 (vNAR_FITC_) was incubated with U87-MG (EGFRvIII^+^/wtEGFR^+^/HER2^−^), HBEC-5i (EGFRvIII^−^/wtEGFR^+^/HER2^−^), and SKBR3 (EGFRvIII^+^/wtEGFR^+^/HER2^+^) cells (Figure 2).

As anticipated, U87-MG cells displayed intense vNAR_FITC_ immunofluorescence. This confirmed robust EGFRvIII-dependent uptake (Figure 2a). No signal was observed in the EGFRvIII-negative control and wtEGFR^+^ HBEC-5i cells (Figure 2b). Notably, SKBR3 cells exhibited a weaker signal than U87-MG cells associated with vNAR_FITC_ specific recognition to EGFRvIII. This suggests potential engagement of low-abundance EGFRvIIII receptor populations in SKBR3 cells (Figure 2c), as also confirmed by Western blot (Figure S2). These findings corroborate the selective internalization of vNAR R426 via EGFRvIII-mediated endocytosis. They also highlight its sensitivity for immunofluorescence detection within the lower EGFRvIII receptor expression profile of HER2-positive breast cancer cells.

### 2.3. vNAR R426 Immunoconjugate Induces Strong Cytotoxicity at Substantially Reduced Cisplatin Doses in SKBR3 Cells

The cytotoxicity of Free cisplatin was initially evaluated in SKBR3 cells across a concentration range of 20–330 µM to establish baseline sensitivity.

**Figure 3:**
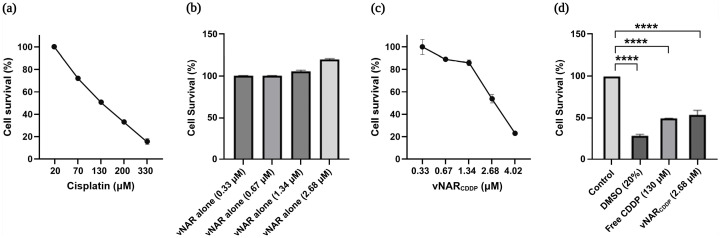
Cell viability of SKBR3 cells after 24 h treatment with vNAR R426-cisplatin immunoconjugate. (**a**) Free Cisplatin (CDDP) dose–response. (**b**) Unconjugated vNAR R426 (vNAR alone). (**c**) vNAR R426-cisplatin immunoconjugate (vNAR_CDDP_) dose–response. (**d**) Comparison of IC_50_ concentrations of free CDDP and vNAR_CDDP_. Data represent SD from three independent experiments. Statistical significance was determined by one-way ANOVA versus the untreated control, followed by Dunnett’s post hoc test (**** *p* < 0.0001).

After 24 h of treatment, the IC_50_ of Free cisplatin was determined to be approximately 130 µM (Figure 3a). To confirm the absence of intrinsic cytotoxicity associated with the targeting vNAR R426 immunoconjugate, SKBR3 cells were exposed to unconjugated vNAR R426 (vNAR alone) at concentrations ranging from 0.33 to 2.68 µM. No reduction in cell viability was observed after 24 h of treatment, indicating that the single domain vNAR R426 itself does not induce cytotoxicity in SKBR3 cells (Figure 3b).

Subsequently, the efficacy of the vNAR R426–cisplatin immunoconjugate (vNAR_CDDP_) in mediating targeted drug delivery was evaluated. SKBR3 cells treated with vNAR_CDDP_ (0.33–4.02 µM) exhibited pronounced, dose-dependent cytotoxicity. Notably, vNAR_CDDP_ achieved an IC_50_ value of 2.68 µM and reduced cell viability to approximately 25% at the highest tested concentration (4.02 µM) (Figure 3c). Importantly, comparison of the IC_50_ values for free cisplatin and vNAR_CDDP_ revealed that vNAR_CDDP_ induced equivalent cytotoxicity at a nearly 50-fold lower cisplatin concentration. These findings demonstrate a substantial increase in cisplatin potency conferred by vNAR conjugation and targeted intracellular delivery (Figure 3d).

These results reveal a substantial enhancement in drug delivery efficiency and cytotoxic potency with the vNAR platform, suggesting that this targeted strategy could enable reduced systemic toxicity and more effective eradication of cancer cells.

### 2.4. Enhanced Cytotoxicity of vNAR_CDDP_ in SKBR3 Spheroids Correlates with Intratumoral Penetration

**Figure 4:**
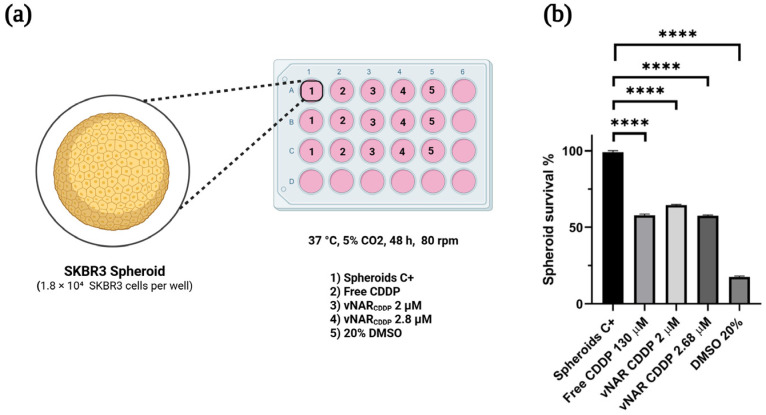
Cytotoxicity of the vNAR_CDDP_ immunoconjugate in SKBR3 spheroids. (**a**) SKBR3 spheroids were generated under orbital agitation for 48 h under standard culture conditions. Twenty spheroids per well were transferred to a fresh plate and treated with free cisplatin, vNAR_CDDP_ or controls for 24 h. Following the treatment, cell viability was assessed using a 4 h Alamar Blue assay on the collected supernatants. (**b**) The treatment with 2.8 µM vNAR_CDDP_ reduced SKBR3 spheroid survival by approximately 50%. Experiments were performed in triplicate. **** *p* < 0.005. Cisplatin (CDDP). Created in BioRender. Lugo, P. (2026) https://BioRender.com/cbpqi5e (accessed on 5 February 2026).

To investigate the therapeutic potential of vNAR_CDDP_ in a physiologically relevant context, we implemented a three-dimensional (3D) SKBR3 spheroids model, each spheroid containing an average of ~900 cells. Then, ~20 spheroids were subculture per well (1.8 × 10^4^ SKBR3 cells) (Figure 4a). Untreated spheroids (Spheroids C+) and a 20% DMSO control served as positive and cell death controls, respectively, while free cisplatin (130 µM, based on previously established IC_50_) and vNAR_CDDP_ at IC_50_ (2.68 µM) and 2 µM were included as experimental treatments (Figure 4b).

Consistent with previous observations in monolayer cultures, vNAR_CDDP_ induced potent, dose-dependent cytotoxicity in spheroids. Spheroid survival assays after 24 h of treatment demonstrated that 2.8 µM vNAR_CDDP_ reduced spheroid viability by ~50%, closely mirroring the response observed in monolayer cultures. Even at 2 µM, vNAR_CDDP_ achieved significant cytotoxicity, while free cisplatin at 130 µM was required to elicit comparable effects. These results indicate that vNAR-mediated delivery enhances cisplatin potency, achieving equivalent therapeutic effects at nearly 50-fold lower drug concentrations in the SKBR3 3D spheroids (Figure 4b) as in monolayer.

### 2.5. EGFRvIII-Specific Binding of vNAR R426 Drives Enhanced Cytotoxicity in SKBR3 Cells

**Figure 5:**
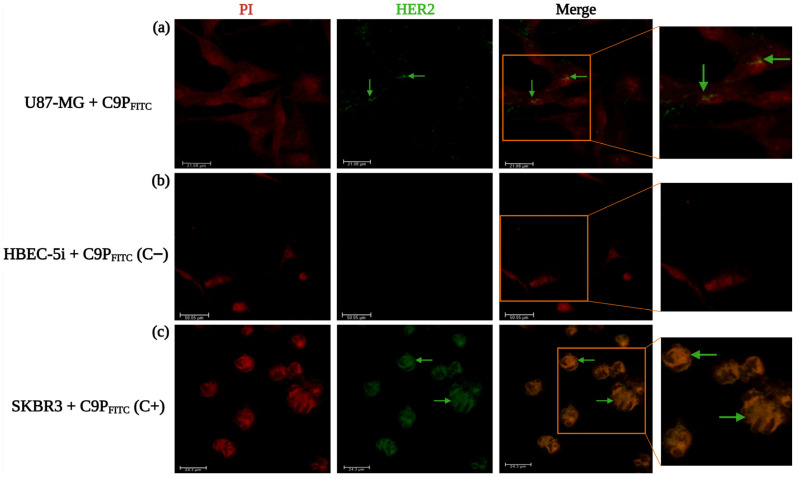
Immunofluorescence analysis of anti-HER2 peptide of FITC-labeled C9P binding and internalization. (**a**) U87-MG cells (EGFRvIII^+^/wtEGFR^+^/HER2^+^), (**b**) HBEC-5i cells (EGFRvIII^−^/wtEGFR^+^/HER2^−^) as negative control, and (**c**) SKBR3 cells (EGFRvIII^+^/wtEGFR^+^/HER2^+^) as positive control for HER2 expression. Cells were incubated with C9P_FITC_ (5.62 µM) for 24 h. Immunofluorescence of C9P is depicted in green and indicated by the green arrows, and nuclei were counterstained with propidium iodide (red). Magnification 40×. Scale bars: 21.08 µm (**a**), 50.5 µm (**b**), 24.3 µm (**c**). Experiments were performed in triplicate.

To determine whether the enhanced cytotoxicity of vNAR_CDDP_ was attributable exclusively to EGFRvIII-specific recognition or potentially to cross-reactive binding to HER2, we employed the previously characterized HER2-targeted peptide C9P labeled with FITC (C9P_FITC_) [25] as a control. Specifically, U87-MG (EGFRvIII^+^/wtEGFR^+^/HER2^+^), HBEC-5i (EGFRvIII^−^/wtEGFR^+^/HER2^−^), and SKBR3 (EGFRvIII^+^/wtEGFR^+^/HER2^+^) cell lines were incubated with C9P_FITC_ (Figure 5a–c).

As anticipated, SKBR3 cells exhibited strong immunofluorescence, consistent with elevated HER2 expression. HBEC-5i cells displayed negligible signal, indicating the absence of HER2 expression and a lack of non-specific binding. U87-MG cells showed minimal C9P_FITC_ signal, as expected for basal HER2 expression in this cell line. In contrast, vNAR_FITC_ produced a comparatively stronger immunofluorescence signal in U87-MG cells relative to C9P_FITC_ (Figure 2a), confirming specific EGFRvIII recognition and engagement, as further validated by Western blot analysis (Figure S2).

Western blot analysis confirmed that EGFRvIII expression is more pronounced in U87-MG cells than in SKBR3 cells (Figure S2). Given that SKBR3 cells are HER2-positive but exhibit lower EGFRvIII expression, these results indicate that vNAR R426 binding is not attributable to HER2 interaction, thereby supporting its specificity for EGFRvIII. The ability of vNAR R426 to efficiently recognize EGFRvIII, mediate cisplatin delivery, and induce cisplatin-mediated cytotoxicity even in cells with lower EGFRvIII expression further underscores its selective engagement of EGFRvIII over HER2 and enhances its therapeutic potential.

### 2.6. Embedded Spheroid Formation and Assessment of vNAR_FITC_ Recognition

**Figure 6:**
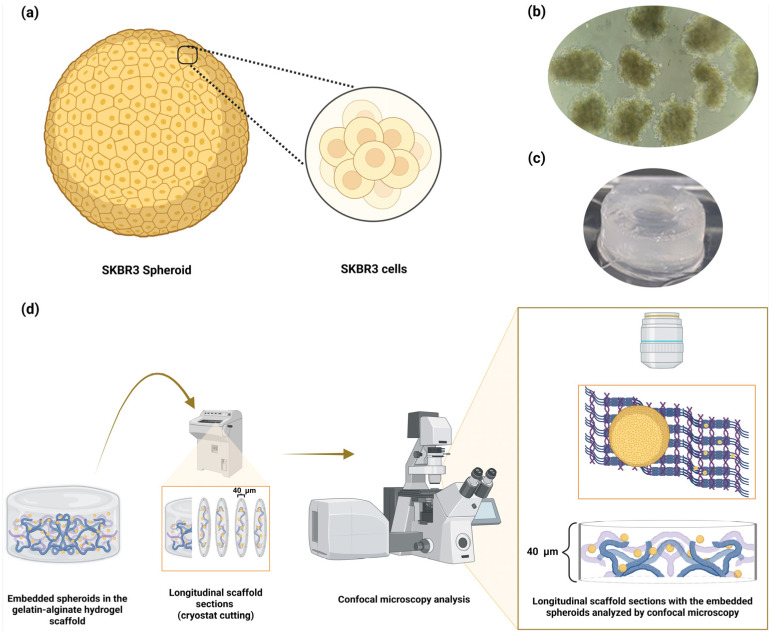
Formation and 3D encapsulation of SKBR3 spheroids for structural assessment prior to vNAR_FITC_, vNAR_CDDP_ and theranostic vNAR (vNAR_CDDP+FITC_) recognition. (**a**) Schematic representation of SKBR3 spheroid cellular organization. (**b**) Optical microscopy image showing SKBR3 spheroid formation after 48 h in 6-well low-adhesion plates (10×). (**c**) Alginate–gelatin matrix containing embedded SKBR3 spheroids following permeabilization. (**d**) Overview of the experimental workflow, illustrating spheroid generation, encapsulation within the 3D alginate–gelatin scaffold, and subsequent microscopy-based evaluation of embedded spheroids. Created in BioRender. Lugo, P. (2026) https://BioRender.com/hj91rr7 (accessed on 5 February 2026).

To evaluate EGFRvIII recognition in a physiologically relevant context, we implemented a 3D spheroid model of SKBR3 cells. Spheroids were formed by a low–adhesion suspension culture and were further embedded in a gelatin–alginate hydrogel scaffold. The embedded spheroids maintained their structural integrity and preserved spatial organization for downstream analysis (Figure 6), following our previous method [26].

After 48 h of culture, individual spheroids contained an average of ~900 cells. We obtained 250–300 spheroids from an initial 250,000 cells. This formation efficiency is consistent with HER2-positive epithelial cell aggregation dynamics and demonstrates the robustness of the low-adhesion culture system. Spheroids exhibited compact and uniform architecture. This reflects cohesive cell–cell interactions, which are essential for evaluating ligand penetration, receptor accessibility, and drug delivery within a three-dimensional tumor-like environment (Figure 6a,b). Following spheroid formation, samples were permeabilized and embedded within the hydrogel matrix (Figure 6c). The experimental workflow is outlined, showing spheroid generation, encapsulation within the 3D alginate–gelatin scaffold, and subsequent microscopy-based evaluation of embedded spheroids (Figure 6d).

### 2.7. vNAR-Based Theranostic Constructs Enable Deep Tumor Penetration and Efficient Intratumoral Drug Delivery in Embedded 3D Spheroid Models

**Figure 7:**
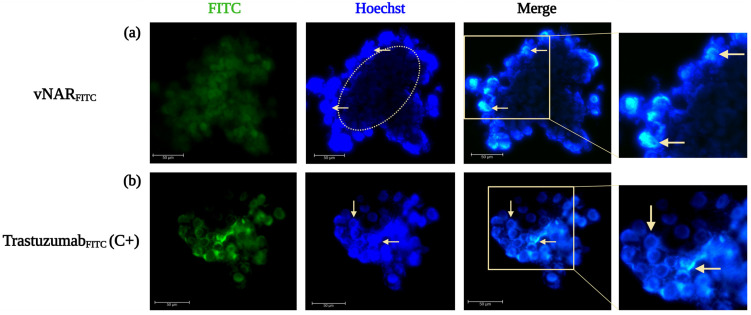
Differential intratumoral penetration of vNAR_FITC_ and Trastuzumab_FITC_ in 3D SKBR3 tumor spheroids at 4 h of treatments. Representative immunofluorescence images of cryosectioned 3D SKBR3 spheroids incubated with vNAR_FITC_ (**a**) or Trastuzumab_FITC_ (**b**). The vNAR_FITC_ exhibits heterogeneous intratumoral and peripheral immunofluorescence, whereas Trastuzumab_FITC_ displays higher overall signal intensity with a more uniform localization, consistent with the limited diffusion of full-length IgG antibodies. The immunofluorescence of vNAR_FITC_ and Trastuzumab_FITC_ is shown in green. Yellow circles and arrows highlight the spatial distribution of vNAR_FITC_ or control; nuclei were counterstained with Hoechst (blue). Scale bars, 27.59 µm (**a**) and 50.23 µm (**b**) Experiments were performed in triplicate. Created in BioRender. Lugo, P. (2026) https://BioRender.com/9wi17cq (accessed on 5 February 2026).

Immunofluorescence detection of cryosectioned tumor spheroids showed efficient, largely heterogeneous distribution and penetration of vNAR_FITC_, with immunofluorescence predominantly localized to the peripheral regions of the spheroid architecture after 4 h of incubation (Figure 7a). In contrast, the FITC-labelled Trastuzumab (commercial therapeutic mAb used for anti-HER2 positive control, Trastuzumab_FITC_) exhibited a more homogeneous distribution (Figure 7b).

The observed differences in spatial distribution are consistent with improved intratumoral diffusion and accessibility associated with the compact vNAR R426 scaffolds in dense three-dimensional tumor microenvironments. The usage of Trastuzumab_FITC_ confirms the HER2^+^ expression in SKBR3. Image acquisition enabled the analysis of immunofluorescence intensity as a function of spheroid depth, providing a spatial profile of ligand distribution from the outer proliferative layers toward the inner hypoxic core.

**Figure 8:**
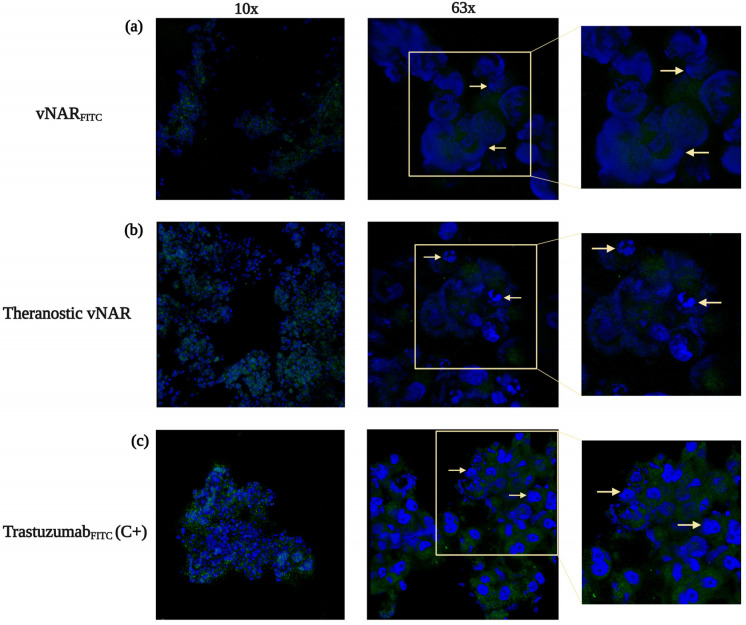
Intratumoral penetration and theranostic distribution of vNAR constructs in embedded 3D SKBR3 spheroids analyzed by confocal microscopy (48 h). (**a**) Representative confocal images of cryosectioned spheroids incubated with vNAR_FITC_ show homogeneous penetration throughout the spheroid. (**b**) Images of spheroids treated with the dual-labelled theranostic vNAR (vNAR_CDDP+FITC_) demonstrate robust intratumoral distribution. Only modest signal attenuation occurs toward the spheroid core. (**c**) Trastuzumab_FITC_ was used as a positive anti-HER2 control. This shows higher overall fluorescence but predominantly peripheral localization, consistent with limited diffusion of full-length IgG antibodies. Immunofluorescence of vNAR_FITC_, theranostic vNAR_CDDP+FITC_, and Trastuzumab_FITC_ is shown in green. Nuclei were counterstained with Hoechst (blue). Yellow arrows indicate cell nuclei. Experiments were performed in a triplicate.

These findings are further supported by confocal analysis of spheroid architecture. Comparable penetration profiles were observed with the vNAR_FITC_ (Figure 8a) and the theranostic vNAR (vNAR_CDDP+FITC_) (Figure 8b), indicating that cisplatin conjugation does not impede tissue diffusion or receptor engagement. Confocal Z-stack imaging revealed robust intratumoral distribution, with only modest attenuation of immunofluorescence toward necrotic central regions (Figure 8a,b), consistent with efficient delivery throughout the spheroid. In contrast, FITC-labelled Trastuzumab (Trastuzumab_FITC_), used as a positive anti-HER2 control, displayed higher overall immunofluorescence but more homogeneous intensity, highlighting the limited intratumoral penetration of conventional full-length antibodies (Figure 8c).

These results are further reinforced in the context of the theranostic vNAR immunoconjugate carrying both cisplatin and a fluorescent reporter (Figure 8b). Immunofluorescence intensity serves as a direct link to receptor-mediated uptake, deep tumor penetration, intracellular distribution, and enhanced cytotoxicity measured in spheroids aligned with these observations. The vNAR_CDDP_ maintained its 50-fold cisplatin concentration reduction while eliminating ~50% of SKBR3 cells (Figure 4b), consistent with early biological effects of effective intracellular drug delivery (Figure 3d). The integration of spatial immunofluorescence profiles with functional viability readouts establishes a robust framework for correlating intratumoral drug distribution with cytotoxicity.

Collectively, these results demonstrate that vNAR R426 functions as an effective theranostic scaffold, integrating deep tumor penetration with potent, targeted cytotoxicity. This work provides a mechanistic rationale for the preclinical evaluation of HER2-positive tumors containing EGFRvIII-positive subclones.

## 3. Discussion

The therapeutic blockade of HER2 has markedly transformed the clinical management of HER2-positive breast cancer; however, significant challenges remain in achieving uniform intratumoral drug distribution and in overcoming resistance mechanisms driven by subclonal heterogeneity and signaling plasticity. Conventional full-length immunoglobulin G (IgG) antibodies and antibody-drug conjugates (ADCs)—including Trastuzumab, Pertuzumab, T-DM1, and Trastuzumab-deruxtecan—are constrained by limited tissue penetration, heterogeneous receptor accessibility, and dose-limiting off-target toxicities [27,28]. Mechanisms of therapeutic escape, such as HER2 downregulation, receptor masking, compensatory signaling, and impaired internalization, further diminish therapeutic efficacy [3]. Even advanced ADCs often display inconsistent payload distribution, off-target toxicity, and limited access to poorly vascularized tumor regions [28].

Despite their widespread clinical use in cancer, autoimmune, and infectious diseases [29,30], full-length antibodies (~150 kDa) largely engage in extracellular targets, restricting access to most intracellular protein–protein interactions [31,32]. Their internalization relies predominantly on endocytosis, often culminating in receptor recycling or lysosomal degradation rather than productive cytosolic delivery [33]. Efficient intracellular targeting requires coordinated receptor engagement, endosomal uptake, and endosomal escape—steps inefficient for conventional IgG-based therapies [34]. While cell-penetrating peptides have been investigated for cytosolic delivery [35], their clinical translation is limited by poor cargo capacity, low cell-type specificity, and in vivo instability [36,37,38,39]. These challenges highlight the need for alternative scaffolds that combine efficient tumor penetration, selective uptake, and functional intracellular delivery.

Single-domain antibodies, such as camelid nanobodies and shark-derived vNARs, offer notable advantages over conventional antibodies, including reduced size, high stability, and robust target specificity. These features translate into improved tissue penetration, more uniform intratumoral distribution, and greater pharmacokinetic flexibility. Nanobody-based drug conjugates have demonstrated enhanced biodistribution and efficacy even in Trastuzumab-resistant HER2-positive tumor models [40,41,42,43,44,45,46], although therapeutic effectiveness against resistant subpopulations remains constrained [3]. Within this landscape, vNAR-based scaffolds represent an emerging strategy for intracellular chemotherapy delivery, potentially redefining the trafficking of payloads in solid tumors.

In this study, we characterized the theranostic potential of the EGFRvIII-specific vNAR R426. Our results demonstrate that specific binding of the vNAR R426 to EGFRvIII compared to wtEGFR (** *p* < 0.0016), equivalent to a commercial monoclonal antibody (** *p* < 0.0026). Also, revealing the selective recognition of EGFRvIII in heat-shock lysates from SKBR3 and U87-MG cells. Collectively, these findings highlight the ability of vNAR R426 to discriminate between mutant and wild-type EGFR receptors, which is a critical prerequisite for targeted cytotoxicity and theranostic applications in heterogeneous HER2-positive tumors.

Functionally, vNAR_CDDP_ exhibited an approximately 50-fold increase in cytotoxic potency compared to free cisplatin; specifically, SKBR3 cells required only 2.68 µM vNAR_CDDP_ to achieve a 50% reduction in cell viability at 24 h, whereas free cisplatin necessitated approximately 130 µM to elicit a similar effect. The absence of cytotoxicity with the unconjugated vNAR further (vNAR alone) confirms that receptor-mediated delivery is the primary mechanism underlying cisplatin-induced cytotoxicity in this context.

The strong correlation observed between intratumoral immunofluorescence and cytotoxicity with the theranostic vNAR immunoconjugate establishes a direct mechanistic association: regions exhibiting high immunofluorescence within SKBR3 spheroids correspond to EGFRvIII-positive subpopulations and display a pronounced therapeutic response, directly linking vNAR distribution to cisplatin therapeutic effect. Administration of vNAR_CDDP_ at 2.68 µM resulted in approximately 50% reduction in spheroid viability, thereby maintaining the 50-fold reduction in cisplatin concentration previously observed in monolayer cultures and confirming that enhanced cytotoxic potency is retained in a physiologically relevant 3D spheroid model.

This effect is further supported by spatial immunofluorescence analysis, which demonstrates that both vNAR_FITC_ and the theranostic vNAR immunoconjugates achieve deep penetration throughout the spheroid, with only modest signal attenuation toward central regions—indicative of efficient receptor-mediated uptake and intracellular delivery. Comparable penetration profiles between cisplatin-conjugated vNAR (vNAR_CDDP_) immunoconjugate demonstrate that payload conjugation does not compromise tissue diffusion or the scaffold’s integrity.

By contrast, FITC-labeled Trastuzumab, used as an anti-HER2 control, displayed higher overall immunofluorescence intensity yet remained largely confined to peripheral spheroid regions, consistent with the known diffusion limitations of full-length IgG antibodies in dense tumor architectures.

Taken together, these findings substantiate the premise that vNAR-based theranostic platforms facilitate a functional linkage between intratumoral distribution and therapeutic efficacy. The integration of immunofluorescence imaging with quantitative cytotoxicity measurements affords spatially resolved insights into receptor engagement, intracellular trafficking, and treatment response, thereby establishing vNAR R426 as a platform with the capacity to enhance drug delivery and surmount diffusion barriers within complex tumor microenvironments.

Mechanistically, the enhanced potency of vNAR_CDDP_ is attributable to EGFRvIII-mediated endocytosis and COPI-dependent retrograde trafficking, which collectively facilitate the nuclear delivery of cisplatin [17]. In contrast to passive cisplatin uptake, this targeted pathway maximizes intracellular drug concentration within therapy-resistant, stem-like EGFRvIII-positive cells [9], while simultaneously intercepting persistent oncogenic signaling that is further amplified by HER2 co-expression [7,8,9,47,48]. The impaired degradation of these receptors results in sustained signaling [7,49], and HER2 heterodimerization promotes oncogenic crosstalk [8]. By directing EGFRvIII through productive intracellular trafficking routes, both vNAR_CDDP_ and theranostic vNAR (vNAR_CDDP+FITC_) constructs achieve dual mechanistic benefits: direct nuclear payload delivery and attenuation of oncogenic signaling. The compact, highly penetrant vNAR-based immunoconjugate enhances receptor accessibility, intracellular accumulation, and uniform payload distribution, thereby overcoming the principal limitations of IgG-based antibody–drug conjugates, which are frequently impeded by endosomal sequestration, receptor recycling, lysosomal degradation, and diffusion barriers [50,51,52,53,54,55,56,57,58].

The modularity of vNAR R426 permits the conjugation of alternative cytotoxins, radiolabels, or imaging agents, thereby enabling the concurrent evaluation of pharmacokinetics, tissue penetration, receptor occupancy, and therapeutic efficacy. Its ability to rapidly diffuse through dense tumor matrices surpasses that of nanobody-based probes such as 2Rs15d and 5F7 [59,60], establishing vNAR R426 as a versatile platform for integrated theranostic strategies. Nonetheless, spheroid models are inherently limited in their ability to fully recapitulate the complexity of in vivo environments, including vascularization, immune surveillance, and stromal heterogeneity [61,62]. Future investigations are warranted to determine pharmacokinetics, biodistribution, stability, toxicity, and to benchmark therapeutic efficacy in vivo.

The vNARs’ unique properties including stability, solubility, and small size grant them with the capability to be suitable for drug conjugation, which yield vNAR-based therapeutics significant advantages for their application in biomedicine, including for targeted drug delivery to solid tumors [17,63] or through Blood–Brain Barrier (BBB) [64]. However, the small size of vNAR-based therapeutics poses a potential challenge in vivo, as rapid clearance via glomerular filtration may limit their circulation time and constrain the therapeutic window [12]. Consequently, strategies to extend the serum half-life of vNARs represent a critical consideration for the development of effective in vivo therapeutics. Notably, human serum albumin (HSA) binding has demonstrated efficacy in prolonging systemic exposure: the anti-HSA vNAR (VNAR-4A12) fused to sfGFP exhibited significantly extended circulation compared to sfGFP alone, without evidence of local or systemic toxicity [65,66].

Another promising strategy to enhance the serum half-life of single-domain antibodies, including vNARs, involves the use of nanoparticles (NP) as controlled drug delivery platforms. These controlled drug delivery vehicles are engineered to enable targeted drug delivery, controlled drug release, and the incorporation of native or recombinant peptide molecules or single domains within an NP matrix, thereby protecting the therapeutic cargo from degradation in harsh biological environments [67,68,69]. In fact, the conjugation of anti-DLL4 vNAR to nanoparticles as therapeutics in pancreatic cancer has been achieved [70]. However, the in vivo pharmacokinetics of these targeted anti-DLL4 vNAR nanoparticles need to be fully elucidated. Nevertheless, vNAR-based therapeutics uphold remarkable potential in targeted cancer nanomedicine, especially in theranostic strategies.

Despite evolutionary divergence from humans (approximately 30% sequence identity with VH/VL domains) [65], vNARs display low immunogenicity, attributable to their compact size and the absence of Fc-mediated effector functions [71,72,73]. Preclinical investigations have demonstrated minimal anti-drug antibody formation and negligible neutralizing activity, even under repetitive or long-term dosing regimens [72,73]. These findings underscore the suitability of vNAR-based immunoconjugates for repeated or long-term therapeutic applications, supporting their potential as minimally immunogenic, tumor-targeted agents—a property particularly advantageous for theranostic platforms like vNAR R426, where sustained delivery and imaging of cytotoxic payloads are critical. Nonetheless, future efforts to address any residual immunogenicity concerns will focus on the humanization of vNARs via the established technique of complementarity-determining region (CDR) grafting, wherein the antigen-recognizing CDRs of vNARs are incorporated into the framework of human VH homologues [74].

In summary, vNAR R426 combines high receptor specificity, efficient intracellular trafficking, potent antitumor activity, and superior penetration of dense tumor architectures. To the best of our knowledge, no analogous vNAR-based immunoconjugates has been reported. The unique characteristic of vNAR R426 facilitates conjugation with alternative cytotoxic payloads and imaging agents, thereby creating a versatile platform for theranostic applications. Collectively, these findings position vNAR R426 as a next-generation immunoconjugate capable of supporting precision oncology strategies by integrating targeted therapy with advanced molecular imaging for EGFRvIII- and HER2-positive breast cancers.

## 4. Materials and Methods

### 4.1. vNAR R426 Protein Expression

The vNAR R426 anti-EGFRvIII was recombinantly produced in *Escherichia coli* BL21 (DE3) cells. Expression used the kanamycin (50 µg/mL) resistant plasmid pET28a+/vNAR R426 [17]. Freshly streaked bacterial colonies (≤1 month stored at 4 °C) were inoculated into 2 × YT medium with kanamycin. Cultures grew overnight at 37 °C with orbital shaking (250 rpm). The pre-culture was then diluted 1:50 into 250 mL of 2 × YT medium and grown to an OD600 ≈ of 0.5. Protein expression was induced with 1 mM IPTG, maintained at 37 °C for 5 h with increased agitation (300 rpm). Cells were harvested by centrifugation (10,000 rpm, 5–10 min), washed with sterile water, pelleted again, and stored at −20 °C until extraction.

### 4.2. vNAR R426 Protein Extraction, Purification, and Quantification

vNAR R426 was extracted under denaturing conditions. Pellets from 200 mL cultures were resuspended in 5 mL sonication buffer (100 mM NaH_2_PO_4_ and 10 mM Tris base, pH 8) and lysed by sonication (500–600 W, 10 s pulses ×4, 40 s rest). After centrifugation (10,000 rpm, 15 min, 4 °C), pellets were resuspended in 4 mL denaturing buffer B (100 mM NaH_2_PO_4_, 10 mM Tris base, 8 M urea, pH 8). The suspension was incubated at 120 rpm for 90 min. Samples were centrifuged (10,000 rpm, 20 min, 20 °C), and the supernatant was incubated with reduced glutathione (GSH, 60 mM final) for 90 min at 120 rpm.

For refolding, the supernatant was diluted into 320 mL of refolding buffer (50 mM Tris base, 5% *v*/*v* glycerol, 0.5 mM oxidized glutathione (GSSG), pH 8.0) and incubated with magnetic stirring for 16 h at 4 °C.

Purification was performed by metal-affinity chromatography on a 5 mL HisTrap HP column (GE Healthcare, Marlborough, MA, USA) using the ÄKTA Pure FPLC system (GE Healthcare, Marlborough, MA, USA).

The sample was loaded at 2 mL/min. Wash steps used wash buffer 1 (50 mM NaH_2_PO_4_, 300 mM NaCl, 70 mM imidazole, pH 8) at 5 mL/min. Elution used a single-step gradient of 300 mM imidazole. The elution buffer contained 50 mM NaH_2_PO_4_, 300 mM NaCl, and 300 mM imidazole at pH 8. Eluted fractions (15 mL) were dialyzed using SnakeSkin Dialysis Tubing (10 kDa MWCO; Cat. No. 68100, Thermo Fisher Scientific, Waltham, MA, USA) against three sequential 0.5 × PBS changes (400 × sample volume; 200 rpm agitation; RT for 2 h per change; final change at 4 °C for 16 h).

Purification quality was assessed by SDS-PAGE (4% stacking gel; 12% separating gel). Western blot validation was performed using an anti-HA–HRP antibody (Cat. No. ab1190; 1:5000 dilution, Abcam, Cambridge, UK). Protein concentrations were quantified using the Micro BCA Protein Assay Kit (Thermo Fisher Scientific, Cat. No. 23235). Quantification was performed using an ELISA microplate format read with the xMark Microplate Spectrophotometer (Bio-Rad, Hercules, CA, USA).

### 4.3. Fluorescein Isothiocyanate (FITC)-Labeled Anti-EGFRvIII vNAR R426

The vNAR R426 anti-EGFRvIII was fluorescently labeled with fluorescein isothiocyanate (vNAR_FITC_; Ex 494 nm/Em 518 nm) using EDC-mediated conjugation. Briefly, vNAR R426 (500 µg) was dissolved in 500 µL conjugation buffer (0.1 M MES, pH 4.5–5.0). It was then mixed with 100 µL EDC solution (1 mg/mL in DMSO) and 500 µg of FITC. The reaction proceeded for 2 h at room temperature. Samples were dialyzed using SnakeSkin Dialysis Tubing (10 kDa MWCO) in 0.5× PBS (pH 3.5–4). Endotoxin removal and concentration were performed with the ProteoSpin Endotoxin Removal Kit (Norgen Biotek, Thorold, ON, Canada) according to the manufacturer’s instructions. The anti-HER2 C9P was fluorescently labeled as a control (C9P_FITC_) using the same protocol.

### 4.4. Immunofluorescence Analyses

U87-MG (ATCC HTB-14), HBEC-5i (ATCC CRL-3245), and SKBR3 (ATCC HTB-30) cells were cultured in their respective media according to ATCC guidelines and maintained at 37 °C in a 5% CO_2_ atmosphere. For confocal microscopic analysis, cells were seeded into 8-well chamber slides at 1 × 10^5^ cells per well (in triplicate). U87-MG cells (EGFRvIII^+^/wtEGFR^+^/HER2^+^) served as the positive control for EGFRvIII expression due to their known EGFRvIII overexpression. HBEC-5i cells (EGFRvIII^−^/wtEGFR^+^/HER2^−^) served as the negative control for null EGFRvIII expression and as a specificity control for wild-type EGFR (wtEGFR^+^) expression. SKBR3 cells (EGFRvIII^+^/wtEGFR^+^/HER2^+^) were included to assess binding specificity and internalization related to HER2 positivity and potential EGFRvIII expression. After a 2 h cell attachment period, experimental treatments—vNAR_FITC_ (0.081 µM/well) or C9P_FITC_ (5.62 µM)—were applied, and cells were incubated for 24 h under standard conditions.

After treatment, the medium was replaced with 4% paraformaldehyde. Cells were fixed for 30 min at room temperature. Slides were washed three times with PBS 1× (pH 7.4) and air-dried. Nuclear counterstaining was performed using propidium iodide (1 mg/mL) for 1 min in the dark. Slides were washed three more times with PBS and air-dried again. Fluorescence imaging was conducted on a Leica SP2 AOBS confocal microscope. The excitation and emission spectra for FITC were set at 494 nm and 518 nm, respectively. For propidium iodide (PI), spectra were collected at 535 nm and 617 nm. All experiments were done in triplicate to ensure reliable signal detection.

### 4.5. vNAR R426 Conjugation with Cisplatin

Cisplatin was conjugated to vNAR R426 (vNAR_CDDP_) using the same EDC method. vNAR R426 (500 µg) was dissolved in 500 µL MES buffer (0.1 M, pH 4.5–5.0). It was mixed with 100 µL EDC solution (1 mg/mL in DMSO) and 500 µg cisplatin (in 500 µL). After 2 h at room temperature, samples were dialyzed with SnakeSkin™ tubing (10 kDa MWCO) against 0.5× PBS (pH 3.5–4). Endotoxins were removed with the ProteoSpin™ kit. vNAR_CDDP_ was concentrated, and protein concentration was measured with the Micro BCA™ Kit in a plate format. Absorbance was read with the xMark™ spectrophotometer (10013301X, Bio-Rad, Hercules, CA, USA).

### 4.6. Theranostic vNAR Generation

To make the theranostic vNAR, vNAR_CDDP_ was conjugated with FITC using the EDC-MES method. Protein amounts were checked using the Micro BCA Protein Kit (23235, Thermo Fisher Scientific) on a plate and read on a xMark spectrophotometer plate reader.

### 4.7. Cell Viability Assays

All cell viability experiments were performed using the AlamarBlue^®^ Cell Viability Assay, following the manufacturer’s protocol. SKBR3 cells (EGFRvIII^+^/wtEGFR^+^/HER2^+^) were seeded at 1 × 10^4^ cells per well in 96-well plates (in triplicate) and incubated for 24 h under standard culture conditions. After each treatment, the culture medium was removed. Cells were incubated with 100 µL of AlamarBlue^®^ solution (1×) (Cat. No. DAL1025, Thermo Scientific) for 4 h. Fluorescence was recorded at 570 nm and 600 nm. Negative controls consisted of untreated cells. Positive controls consisted of cells exposed to 20% DMSO. The 20% DMSO is an effective positive control for cell death in viability assays (e.g., MTT, trypan blue) when spheroids were used. The DMSO diffusion was affected by spheroid size, presence of extracellular matrix (EMC), and perfusion to spheroid center [75]. In our essays, the gelatin–alginate solution used for SKBR3 spheroid embedded could simulate the EMC barrier affecting DMSO perfusion. In consequence increments in DMSO concentration achieve cell death and reduces metabolic activity by more than 30%, serving as a robust standard for 100% toxicity as previous report [76,77]. All assays were performed in triplicate.

#### 4.7.1. Cell Viability Cisplatin Assay in SKBR3 Cell Line

After 24 h, SKBR3 cells were treated with different cisplatin doses (20–330 µM) for 24 h. After treatment, the medium was removed, and AlamarBlue solution (100 µL) was added for 4 h. Absorbance was checked at 570 and 600 nm. Negative and positive controls matched those in Section 4.7.

#### 4.7.2. Cell Viability Assay with vNAR 426 Alone

To test if vNAR alone was toxic, SKBR3 cells were treated with vNAR R426 doses (0.33–2.68 µM) for 24 h. After the medium was taken out, 100 µL AlamarBlue solution was added for 4 h, and absorbance at 570 and 600 nm was measured. Controls and repeats followed by Section 4.7.

### 4.8. Cell Viability Assay with Cisplatin-vNAR R426 Immunoconjugate in Monolayer

To determine the cytotoxic potency of the cisplatin-conjugated vNAR, SKBR3 cells (EGFRvIII^+^/wtEGFR^+^/HER2^+^) were seeded at 1 × 10^4^ cells per well (triplicate) and incubated for 24 h in a monolayer. Then, they were treated with vNAR_CDDP_ at concentrations ranging from 0.33 to 2.68 µM for an additional 24 h. Following treatment, the medium was replaced with 100 µL AlamarBlue^®^ solution and incubated for 4 h. Absorbance was measured at 570 and 600 nm. Negative and positive controls were included as previously described.

### 4.9. Cell Viability Assay in 3D SKBR3 Spheroids

SKBR3 cell spheroids were generated using a suspension culture method in 6-well ultra-low attachment plates to emulate a three-dimensional tumor microenvironment. Briefly, 2.5 × 10^5^ SKBR3 cells, previously expanded to ~90% confluence in T25 flasks with 98% viability, were seeded per well in RPMI medium supplemented with 5% fetal bovine serum (FBS) and 1% antibiotic solution. Cultures were maintained under orbital agitation (80 rpm) in a humidified incubator at 37 °C with 5% CO_2_ for 48 h to promote spheroid aggregation.

Following incubation, 20 spheroids were transferred to 24-well ultra-low attachment plates (1.8 × 10^4^ cells per well), to demonstrate vNAR_CDDP_ penetration and enhancement of cytotoxicity within the 3D SKBR3 spheroids. Treatments were performed in triplicate using an additional concentration of vNAR_CDDP_ (2 µM) and the previously established IC_50_ concentrations: cisplatin (Free CDDP, 130 µM) and the vNAR_CDDP_ (2.68 µM). Untreated spheroids (Spheroids C+) served as positive survival control, and a 20% DMSO condition was included as cell death control.

After 24 h of treatment, 10% (*v*/*v*) resazurin solution was added to each well and incubated for 4 h under standard culture conditions. Cell viability was quantified using the Alamar Blue assay, measuring supernatant absorbance at 570 nm and 600 nm with a microplate reader. The data was analyzed with One-way Anova with Sidak’s Multiple comparison test (**** *p* < 0.0001).

### 4.10. Gelatin–Alginate Scaffolds and Spheroid Formation

To confirm vNAR penetration in homogeneous spheroids, scaffolds containing SKBR3 cell spheroids were prepared using a previously reported methodology [26]. A 7.5% gelatin and 3.75% alginate mixture was prepared, and 800 µL of the solution was dispensed into the center of circular silicone molds. Samples were incubated at 4 °C for 30 min to allow initial gelation. The scaffolds were then demolded and crosslinked by adding 500 µL of 150 mM CaCl_2_ for 30 min. After crosslinking, scaffolds were washed three times with 1× PBS (5 min each) and transferred into 24-well plates for subsequent experiments.

#### 4.10.1. Spheroid Formation

Spheroids were made by culturing cells in low-adhesion plates. SKBR3 cells grew in T25 flasks to about 90% confluence and 98% viability. 250,000 cells were added to each well of 6-well low-attachment plates with RPMI medium plus 1% antibiotic and 5% fetal bovine serum. Cultures were incubated for 48 h at 80% humidity, 5% CO_2_, and 37 °C, with gentle shaking at 80 rpm to promote spheroid growth.

Cell counting was performed via optical microscopy. To count the cells, individual spheroids were carefully aspirated with a micropipette, transferred to 1.5 mL tubes, and dissociated with 50 µL of trypsin–EDTA for 10 min. Following complete disaggregation, cells were stained with trypan blue and counted using a Neubauer chamber.

#### 4.10.2. Spheroid Encapsulation in Scaffolds After Treatment

After 48 h of spheroid formation, a final concentration of 20 µM of each treatment (vNAR_FITC_, theranostic vNAR [vNAR_(CDDP+FITC)_], C9P_FITC_, or Trastuzumab_FITC_) was added per well and incubated according to the experimental treatments. Spheroids were incubated for an additional 24 h under standard culture conditions. The culture medium was then replaced with 4% formaldehyde, and the samples were fixed for 30 min at room temperature. After fixation, formaldehyde was replaced with 50% ethanol for 30 s, followed by gentle washing with 1× PBS. Each well was replenished with 100 µL of supplemented medium. Treated spheroids were then collected and transferred into silicone molds containing 800 µL of the gelatin–alginate mixture. The encapsulation followed the methodology described in Section 4.9, with the addition of 30% sucrose at this stage. Samples were subsequently incubated for 72 h at 4 °C to complete scaffold stabilization.

#### 4.10.3. Cryosectioning of Encapsulated Spheroids

After 72 h, encapsulated spheroids were transferred to cryomolds and embedded in thin layers of cryostat embedding medium (Tissue-Tek, 4583) until fully surrounded and frozen. Cryosections 40 µm thick were obtained using a cryostat (Leica CM1860, Leica Biosystems, Deer Park, IL, USA) set to −25 °C. Sections were mounted onto standard microscope slides pre-coated with 2% gelatin to prevent sample detachment during staining.

#### 4.10.4. Nuclear Staining of Encapsulated Spheroids

Cryosections were stained with Hoechst 33258 following the manufacturer’s instructions and incubated for 10 min at room temperature. Slides were washed twice with 1× PBS and allowed to air-dry in the dark. Immunofluorescence imaging was performed using a Leica DMi1 microscope in sequential acquisition mode to minimize background interference. Image analysis was carried out using Leica LAS X software version 3.0.0.15697.

### 4.11. Statistics

One-way ANOVA and Dunnett’s post hoc test were used to check statistical significance, set at *p* < 0.05. Detailed descriptions of the specific analyses for each experiment are provided in the corresponding Methods sections. All analyses were done with GraphPad Prism 10 version 11.0.0.

## 5. Conclusions

Our study establishes vNAR R426 as a compact, high-affinity single-domain scaffold with dual therapeutic and immunofluorescent capabilities in HER2-positive breast cancer. Conjugation to cisplatin (vNAR_CDDP_) mediates potent, receptor-specific cytotoxicity in SKBR3 cells and SKBR3 spheroids at an IC_50_ of 2.68 µM, representing a ~50-fold increase in efficacy relative to free cisplatin. The theranostic vNAR immunoconjugate preserves these effects while enabling simultaneous visualization of tissue penetration and receptor engagement, confirming that payload conjugation does not compromise specificity, diffusion, or intratumoral distribution.

In the 3D SKBR3 spheroids, vNAR_FITC_ and theranostic vNAR immunoconjugates achieved heterogeneous yet deep penetration, effectively reaching inner cell populations typically inaccessible to conventional IgG antibodies. This demonstrates the vNAR 426-based immunoconjugates’ capacity for precise intracellular delivery and highlights the advantage of its small size and modularity for versatile functionalization and integrated theranostic applications. To our knowledge, no analogous vNAR-based immunoconjugates have been reported, underscoring its novelty.

Collectively, these results establish vNAR R426 as a unique receptor-specific theranostic platform, coupling efficient intracellular drug delivery with spatially resolved immunofluorescence, and providing a robust foundation for next-generation precision oncology strategies targeting HER2-positive tumors with EGFRvIII-positive subclones.

## Data Availability

The original contributions presented in this study are included in the article/Supplementary Material. Further inquiries can be directed to the corresponding author(s).

## Supplementary Materials

The following supporting information can be downloaded at: https://www.mdpi.com/article/10.3390/ph19040633/s1, Figure S1: Recognition ELISA for vNAR R426 against pEGFRvIII peptide and comparison with wtEGFR; Figure S2: Western blot of heat-shock lysate of SKBR3 and U87-MG cells, comparing recognition of commercial monoclonal antibody with the vNAR R426; Table S1: Absorbance spectra (190–220 nm) of the immunoconjugates; Table S2. vNARCDDP relative concentration based on AUC. References [78,79,80,81,82,83,84] are cited in the Supplementary Materials.
