# Supplementary material for: Theranostic vNAR-Based Immunoconjugates Achieve Selective Intracellular Cisplatin Delivery in Embedded 3D HER2-Positive Breast Cancer In Vitro Model
Source: Pharmaceuticals (Basel). 2026 Apr 17;19(4):633. doi: 10.3390/ph19040633 (PMC13119102; doi:10.3390/ph19040633)
Supplement: Supplementary file 1 [file pharmaceuticals-19-00633-s001.zip › pharmaceuticals-4169277-supplementary.pdf]

Supplemetary Materials: Theranostic vNAR-based immunoconjugates achieve selective intracellular cisplatin delivery in embedded 3D HER2-positive breast cancer in vitro model

Andrea C. Alfonseca-Ladrón de Guevara, Alejandro Manzanares-Guzmán, Jessica Badillo-Mata, Mirna Burciaga-Flores, Pavel H. Lugo-Fabres and Tanya A. Camacho-Villegas

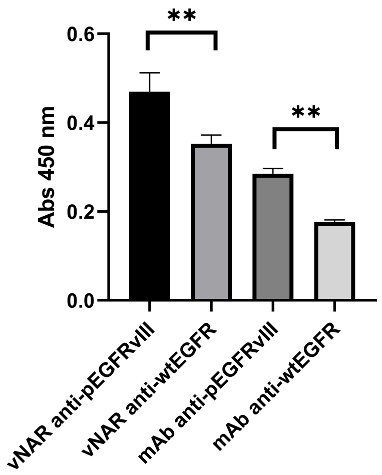

Figure S1. Recognition ELISA for vNAR R426 against pEGFRvIII peptide and comparison with wtEGFR. The hatched bars represent the specific recognition of vNAR R426 to the EGFRvIII receptor. Anti-pEGFRvIII and Anti-wtEGFR were used as controls. A statistically significant difference was observed between vNAR R426 recognition of pEGFRvIII and wtEGFR (** $p < 0.0016$). Similar results were observed using a commercial monoclonal antibody (MyBioSource, MBS9461087), which recognized pEGFRvIII significantly better than wtEGFR (** $p < 0.0026$).

1.2 Western blot comparing recognition of heat-shock lysate of SKBR3 and U87-MG cell lines

The molecular weight of the EGFRvIII protein is 145 kDa, and we demonstrated that the vNAR R426 can recognize the monomeric form of the receptor (Figure S2, right panel, black arrow). The 145 kDa band is more prominent in U87-MG cells than in SKBR3 cells. Also, it is possible that the ~45 band shown in vNAR R426 corresponds to cross-reactivity with a new type of EGFR, as reported by Piccione et al., [80], called mini-LEE (mLEEK). Moreover, Minileek (mLEEK) is a 45 kDa variant of the epidermal growth factor receptor (EGFR) that has been identified in U87MG human glioblastoma cells [80]. In contrast, the commercial mAb detects only bands at ~50 and ~80 kDa, possibly due to EGFRvIII truncation variants. This sustains our results shown in the manuscript.

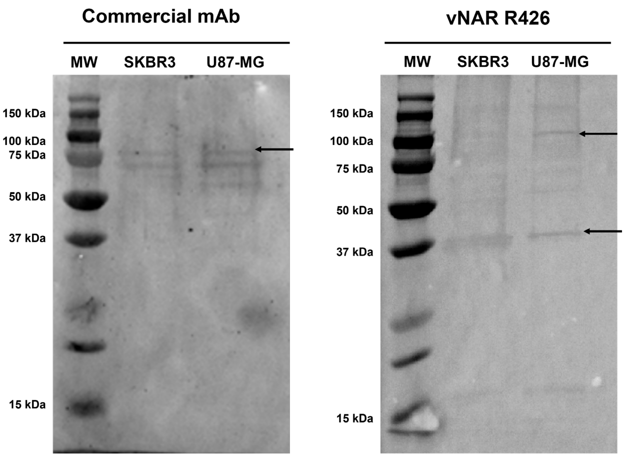

Figure S2. Western blot of heat-shock lysate of SKBR3 and U87-MG cells, comparing recognition of commercial monoclonal antibody with the vNAR R426. MW: Molecular weight marker. The heat-shock lysate corresponds to 1 million cells in stock (SKBR3 or U87-MG). One membrane was probed with a commercial mAb (left), and the other with vNAR R426 (right). The vNAR recognizes bands on 145 and ~45 kDa, indicated with arrows. The mAb recognizes only an ~80 kDa protein.

1.3 Relative quantification of vNAR R426-CDDP conjugation

The conjugated sample vNAR$_{CDDP}$ exhibited a modified absorbance profile compared with the spectra of the individual components, consistent with the formation of a drug–protein complex. consistent with the formation of a drug–protein complex. The area under the curve (AUC) in the 190–220 nm range (**Table S1**) was calculated using the trapezoidal method **(Table S2**). The vNAR–cisplatin (vNAR$_{CDDP}$) conjugate showed an AUC value of 17.12, whereas cisplatin alone presented an AUC of 23.04. Using Equation 1, the approximate Drug-to-Protein Ratio (DAR) was determined to be ~1.34 [80]. The DAR of 1.34 was a positive result, given the vNAR size, the tendency of agglomeration in conjugated protein, and possible allosteric impediment. We hypothesize that the DAR of 1.34 for vNAR R426 prioritizes cell penetration and nuclear drug delivery, as shown in Figure 3d, where vNAR R426 reduces SKBR3 viability by ~50-fold compared to free cisplatin. Based on the DAR result and considering two hypothetical conjugation sites for each vNAR R426, the conjugation efficiency was estimated to be approximately 67.5%.

Table S1: Absorbance spectra (190–220 nm) of the immunoconjugates.

Wavelength (nm)	CDDP	vNAR	vNAR$_{CDDP}$	FITC	vNAR$_{FITC}$	vNAR-$_{FITC-CDDP}$
**190**	0.019	0.436	0.027	0.277	0.011	0.00
**200**	1.037	0.331	0.731	0.717	0.487	0.278
**210**	1.030	1.542	0.837	0.551	0.314	0.094
**220**	0.455	1.446	0.261	0.458	0.171	0.025

Table S2. vNAR$_{CDDP}$ relative concentration based on AUC.

Sample	AUC (190–220 nm)
**Cisplatin**	23.04
**vNAR**	28.14
**vNAR$_{CDDP}$**	17.12

2. Materials and Methods

2.1 EGFRvIII peptide recognition ELISA assay

To demonstrate specific recognition of vNAR R426 against the EGFRvIII peptide (pEGFRvIII), a final concentration of 500 ng per well was immobilized in an ELISA plate. As control of recognition, the same concentration was used well with native EGFR (wtEGFR). All assays were in triplicate. Both (EGFRvIII and wtEGFR) antigens were used to compare the results with a commercial antibody declared as specific for mutated and wtEGFR.

The plate was incubated for 12 h at 37 °C; the wells were blocked with 150 μL of 3% BSA-1X PBS for 2 h at 37°C. Then, decanted and washed three times with PBST. In wells with immobilized receptors pEGFRvIII (Peptide 2.0, batch 177089-00L) and wtEGFR (PeproTech, 100-15R-10UG) the vNAR R426 was used with a final concentration of 28 μg and incubated for 4 h at 37 °C. Then, 50 μL of anti-His-HRP was diluted 1:3,000 in 1% BSA-1X PBS and incubated for 1 h at 37 °C.

For wells when the commercial anti-EGFRvIII-HRP antibody (MyBioSource, MBS9461087) a dilution of 1:1000 was used and incubated at standard conditions. Finally, 50 μL of TMB ELISA substrate was added (Thermo Scientific, 34028) for wells and incubated for 15 min at 37 °C. The reaction was stopped with 50 μL of 2 M sulfuric acid. The absorbance was detected at 450 nm in a Benchmark Microplate Reader (BIORAD, 170–6850) and plotted. The data were analyzed with a one-way ANOVA and a post hoc Bartlett test.

2.2 Western blot comparing heat-shock lysate of SKBR3 and U87-MG cell lines

To demonstrate the recognition of EGFR receptors in U87-MG ($EGFRvIII^+/wtEGFR^+/HER2^+$) and SKBR3 ($EGFRvIII^+/wtEGFR^+/HER2^+$) cell lines, we performed a western blot from heat-shock lysate cells using the vNAR R426 and a commercial monoclonal antibody as control. Briefly, 1x106 cells were centrifuged at 5000 xg for 5 min, washed with sterile 1X PBS and resuspended in 100 μL of nuclease-free water. Then, tubes heated at 80 °C for 10 min with vortex every 2 min to sample homogenization and centrifuged in the same conditions. The supernatant was recovered and precipitated using a cold 100% TCA (15% in tube) incubated overnight at –80 °C. Then washed two times with cold acetone, centrifugated and resuspended in 100 μL 2X Laemmli SDS sample buffer denaturing at 90 °C for 5 minutes. A 12% SDS-PAGE gel was used to characterize the protein content loading 20 μL of heat-shock lysate for each cell line. Two gels were used with the same order: MW ladder (BioRAd, 1610375), SKBR3 lysate ($EGFRvIII^+/wtEGFR^+/HER2^+$) and U87-MG lysate ($EGFRvIII^+/wtEGFR^+/HER2^+$). Each gel was transferred to the nitrocellulose membrane for 60 minutes at 200 mA using the semi-dry transfer protocol (Bio-Rad TRANS-BLOT SD transfer system). After blocking the membrane with 3% BSA - 1X PBS for 1 h, one membrane was incubated with vNAR R426 as primary antibody (1.5 μg/mL - 0.10 μM) and incubated overnight at 4 °C. Then, this membrane was washed five times (PBST) and incubated with anti-His-HRP as secondary antibody at 1:1500 dilution (Roche, 11965085001), for 2 h at 37 °C. For the second membrane, the commercial anti-EGFRvIII-HRP antibody (MyBioSource, MBS9461087) was used at 1:1000 dilution. Then incubated at 4 °C overnight. Finally, both membranes were incubated with DAB (3, 3′ diaminobenzidine tetrahydrochloride) substrate until color development. Images were obtained using a Bio-Rad GelDoc Go imaging system.

2.3 Relative quantification of vNAR R426-CDDP conjugation

UV–Visible spectroscopy was used to evaluate the interaction between cisplatin and the vNAR antibody fragment. Absorbance spectra were recorded in the 190–220 nm range considering the characteristics of absorption peak located at 207 nm [82]. For this evaluation, we include cisplatin (CDDP), vNAR R426, vNAR conjugated with cisplatin ($vNAR_{CDDP}$), FITC, $vNAR\text{-}_{FITC}$, and $vNAR\text{-}_{FITC\text{-}CDDP}$. The area under the curve (AUC) was calculated using Equation 1 [83, 84].

***Equation S1*:**

$$Conjugation\ signal = \frac{AUC_{CDDP}}{AUC_{vNAR\ CDDP}}$$
